# Can We Predict Renal Function Recovery After Pyeloplasty in Pediatrics with Ureteropelvic Junction Obstruction? A Systematic Review

**DOI:** 10.5152/tud.2024.23220

**Published:** 2024-03-01

**Authors:** Safendra Siregar, Akhmad Mustafa, Steven Steven

**Affiliations:** 1 Department of Urology, Hasan Sadikin Academic Medical Center, Universitas Padjadjaran, Bandung, Indonesia

**Keywords:** Chronic unilateral renal obstruction, pyeloplasty, predictors

## Abstract

Chronic unilateral renal obstruction, primarily caused by ureteropelvic junction obstruction (UPJO), poses challenges in determining the optimal timing for corrective surgery. The goal is to preserve renal function and alleviate symptoms, but there is no definitive diagnostic test to reliably predict the outcomes of surgery. This systematic review aimed to identify predictors for renal function recovery after pyeloplasty in order to guide effective treatment options. We conducted a systematic review following the Preferred Reporting Items for Systematic Reviews and Meta-Analysis guidelines. A literature search was performed on PubMed, Embase, and Scopus using keywords related to renal function, pyeloplasty, and predictors. The search was conducted on March 10, 2022. The quality of the included studies was assessed using the Newcastle–Ottawa Scale. Out of 344 potentially relevant articles, 11 met the eligibility criteria for this study. These included 6 retrospective and 5 prospective studies, with a total of 925 participants. Most studies evaluated renal function using differential renal function (DRF). The overall quality of the included studies was considered average. The findings indicated that age at the time of surgery and gender did not significantly influence functional recovery after pyeloplasty. However, preoperative DRF consistently emerged as a critical predictor. Preoperative DRF can serve as the most common predictors used for renal function recovery following pyeloplasty. These findings contribute to understanding effective treatment options for chronic unilateral renal obstruction. However, further research for each predictor is needed to validate these predictors and their clinical utility.

## Introduction

Chronic unilateral renal obstruction is more frequently caused by ureteropelvic junction obstruction (UPJO). Preserving renal function from deteriorating and relieving symptoms are the treatment goals for UPJO patients.^1,2^ Literature has reported that patients with lower renal function preoperatively were less likely to improve, and differential renal function (DRF) would unlikely improve after the alleviation of the obstructions.^3,4^

Treatment for UPJO is still debatable, owing to the confusion regarding postoperative renal function recovery.^5^ Based on literature, pyeloplasty is indicated for patients with reasonable DRF rather than a poorly functioning kidney (PFK) with a cutoff <10%.^6^ The current diagnostic examinations, such as ultrasonography, biological markers, magnetic resonance imaging, and drainage or the split function on the diuretic scan, cannot be utilized to predict renal units that might have benefits of surgery from preserved or improved renal function; therefore, the timing to surgically correct UPJO (presumed) is still controversial.^7^ As a result, experts in urology and nephrology use preoperative or management predictors to help in decision-making about conducting nephrectomy or pyeloplasty.^8^ This study’s objective was identifying predictors for the capability of renal function recovery following a pyeloplasty.

## Materials and Methods

### Eligibility Criteria

This systematic review includes studies if they met the following criteria: (1) published in English and available in full-text, (2) published up to January 2022, (3) the studies were randomized controlled trials (RCTs) or observational studies, (4) participants were below 18 years old (pediatric), (5) underwent pyeloplasty, (5) the studies reported the predictors of successful pyeloplasty. We exclude studies that did not include differential renal function (DRF) as preoperative and postoperative renal function evaluation.

### Guidelines

**Figure 1.**
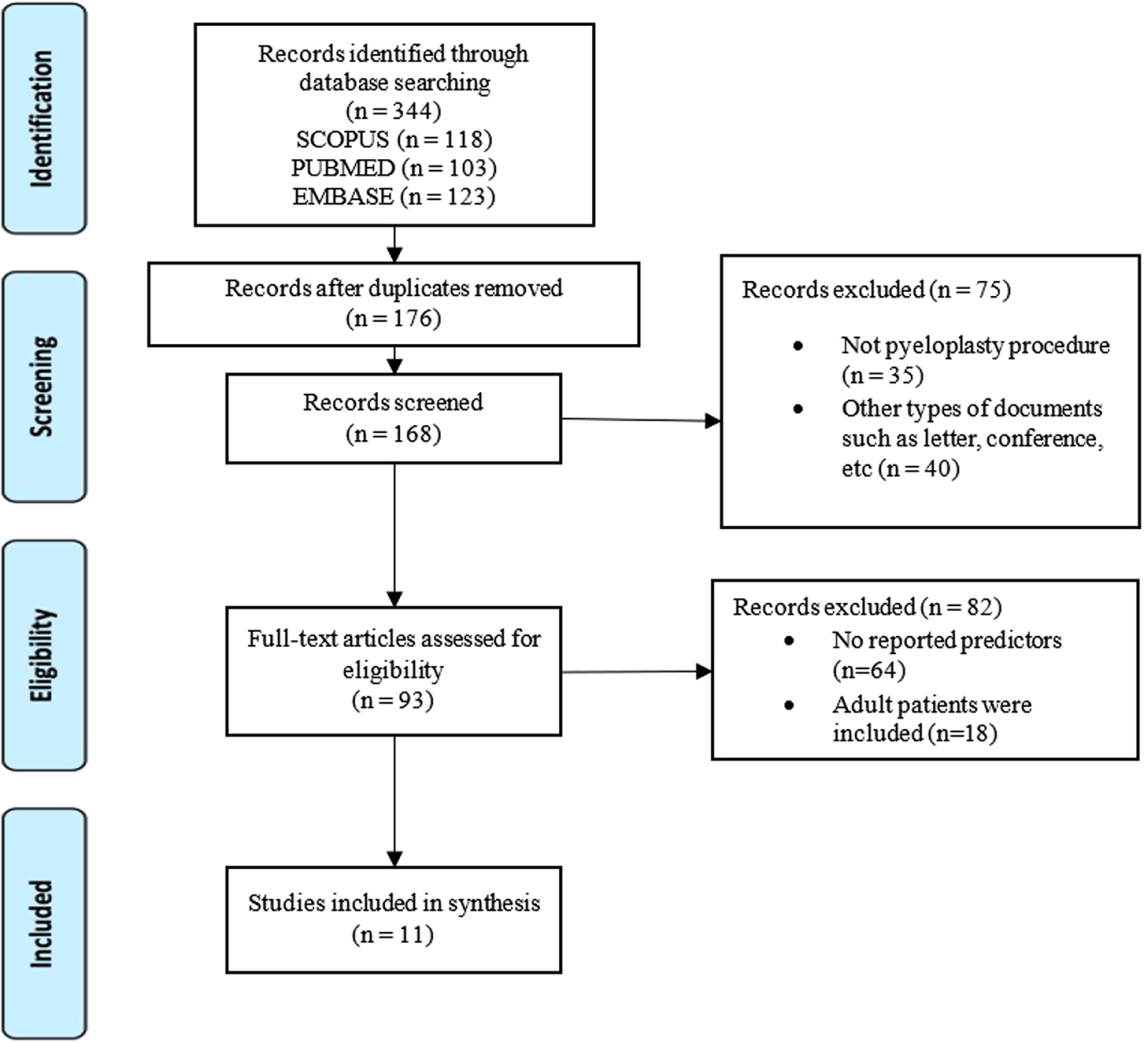
Study selection using Preferred Reporting Items for Systematic Reviews and Meta-Analyses flow diagram.

This study used the Preferred Reporting Items for Systematic Reviews and Meta-Analysis (PRISMA) scale.^9^ The flow diagram is shown in Figure 1.

### Search Strategy

Following PRISMA guidelines, we conducted a literature search on PubMed, Embase, and Scopus. We performed literature search on March 10, 2022, with terms including (“renal” AND “function” AND “pyeloplasty” AND (“predict*” OR “factor*”)). We followed the Cochrane Handbook for systematic reviews of interventions guidelines for the search.^10^

### Quality Assessment and Risk of Bias

Case–control and prospective cohorts were evaluated using the Newcastle–Ottawa Scale for their quality. The studies are judged based on 3 standpoints: study group selection, comparability between groups, and the assessment of exposure and outcome of interest. For bias evaluation, low risk is scored as ≥7, moderate as between 4 and 6, and high risk as ≤3.^11^

### Data Extraction

One reviewer selected literature and extracted data into an Excel database. Titles and abstracts screening were conducted to determine the eligible articles by 2 authors. Then, a full-text review was performed to find detailed information. Data were extracted by 2 authors independently. Information on the author, study design, publication year, population, sample size, age, type of procedure performed, predictor factors, diagnostic modalities, and renal function tests were extracted to MS Excel. Any disagreements were thoroughly discussed until an agreement was finally reached between the observers.

## Results

### Study Selection

In total, 344 potential articles were included from the systematic search of all databases (Figure 1). We examined and excluded duplicates, leading to 176 articles. Furthermore, a total of 93 articles were eligible for this study. We included 11 articles following full-text reviews.

### Study Characteristics

The study characteristics are shown in Table 1. Six studies consisted of retrospective, and 5 were prospective in study design. The number of included participants in each study varied, with a total of 925 male-to-female ratios of 2.5 : 1.

Nine studies used Anderson–Hynes dismembered pyeloplasty technique, and 1 study used both dismembered and non-dismembered techniques for comparison. Three out of 11 studies did not mention the side of pyeloplasty, and 7 out of 8 remaining studies reported a higher number of left-sided pyeloplasty than right one.

Most studies evaluated renal function using DRF and glomerular filtration rate (GFR). Diethylenetriamine pentaacetate (DTPA) was used more than mercaptoacetyltriglycine (MAG3) and Tc-99m dimercaptosuccinic acid (DMSA). All the studies considered increased DRF > 5% as significant renal recovery after surgery.

### Quality Assessment

**Figure 2.**
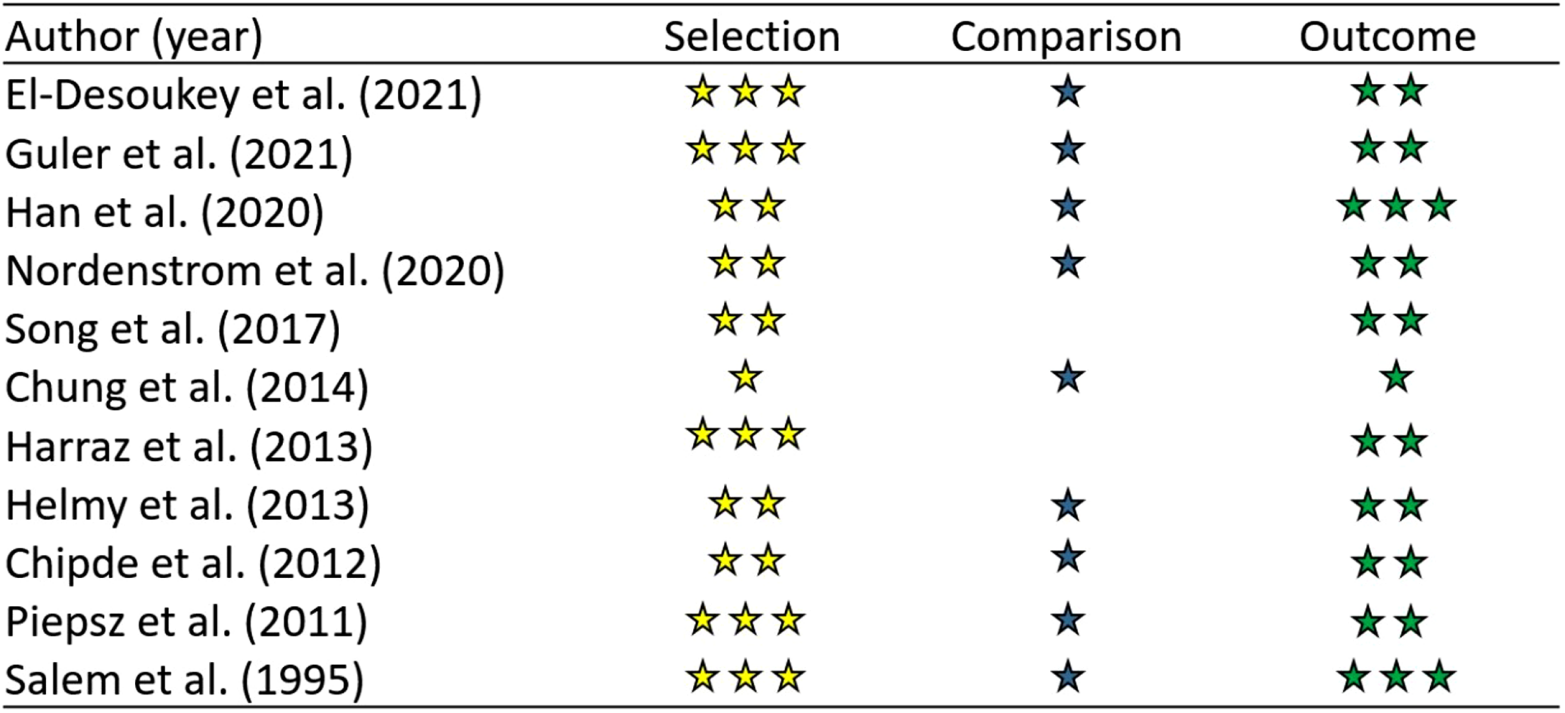
The risk of bias assessment using the Newcastle–Ottawa scale.

We evaluated the quality and risk of bias by using Newcastle–Ottawa Scale.^11^ We evaluated each study and recap each of them as seen in Figure 2. The quality was considered average for the included studies. One study was considered low risk for bias, 9 had a moderate bias, and 1 had a high risk of bias. Therefore, the result showed a relatively moderate chance of bias. A meta-analysis of this study could not be conducted because the collected studies were heterogeneous, including the isotype used for DRF evaluation and study population.

### Renal Function Test

Nowadays, multiple laboratory workups are available to investigate and evaluate kidney function. Clinically, renal function is most commonly assessed using the GFR estimation modality.^12^ In this review, several methods were used to evaluate renal function, consisting of DRF or split renal function (SRF), estimated glomerular filtration rate (eGFR), and relative renal function.

All studies used DRF or SRF to compare the pre- and post-pyeloplasty renal function in patients with UPJO. Split renal function (DRF) is 1 kidney’s contribution relative to the total renal function. It is useful to evaluate and guide treatments for various renal disorders. Differential renal function can be evaluated using renal scintigraphy with radionuclides such as ^99m^Tc-DMSA, ^99m^Tc-MAG3, and ^99m^Tc-DTPA.^13^ Decreased split function is considered a functional deterioration of the kidney, although the split function can also be decreased without deterioration of the kidney’s absolute function. A study by Piepsz et al^14^ showed that decreased split function was not associated with decreased single kidney glomerular filtration rate.

The second method used by studies eGFR Glomerular filtration rate is crucial in assessing renal function in clinical practice, research, and published health practice. The reference standard is the measured GFR, although major advances in eGFR have been shown in the last 20 years.^15^

Additional methods consisted of the renal resistive index, residual renal function, and renal parenchymal volume. The renal resistive index is a prognostic marker in vascular diseases affecting the kidney, which is nonspecific. It has been proposed that the renal resistive index, as determined by duplex ultrasonography, is a good predictive tool for identifying individuals who would not benefit from revascularization in terms of improved renal function or blood pressure. A high score for resistive index (>0.8) in native kidneys is related to kidney dysfunction and adverse cardiovascular effects.^16^ In patients who underwent renal surgery, decreased renal function in a few months following the surgery is best associated with decreased parenchymal volume of the kidney. Volume loss estimation can be beneficial in predicting postoperative renal function in those who will undergo surgical intervention with a solitary kidney.^17^

### Predictive Factors for Renal Function Improvement Post Pyeloplasty

Studies in this review discovered several predictors for renal function recovery post pyeloplasty (Tables 2, 3 and 4). The first factor was the age at diagnosis and surgery. A study by Nodenstrom et al^18^ suggested that antenatally-detected patients had a better DRF improvement or catch-up. This result is more interesting because more studies have shown no difference between the recovery of renal function based on age at diagnosis (pre and postnatal).^19-21^ Salem et al^22^ showed that the overall likelihood of reduced or improved preoperative DRF becoming reduced or improved in the postoperative period is not affected by age. All studies that explored the correlation between gender and results of pyeloplasty found that no association exists between the 2.^19-21,23-25^

The most crucial factor affecting postoperative recovery was shown to be preoperative DRF (Table 2). Studies stated that in patients with a preoperative DRF of under 40%, the patients’ DRFs were more likely to improve.^18,21,22,26^ Harraz et al^21^ stated that patients with lower preoperative DRF have more room for kidney recovery than those with a better DRF. Besides DRF, classic conventional markers of renal function such as serum creatinine can be used. Chipde et al^27^ also found that recovery could be predicted based on the ratio of protein and creatinine (Pr/Cr) from urine obtained from the renal pelvis. Renal pelvis’s Pr/Cr of over 0.5 can be found in all patients with improved DRF after pyeloplasty. Recent study with proteomics has become more important in diagnostic and prognostic tools. Using proteomic analysis, researchers found several other biomarkers of renal injury and dysfunction, potentially predicting the prognosis and treatment of children with PUJO.^28^

The anteroposterior diameter (APD) of the pelvis was one of the predictors for pyeloplasty (Table 3). Four studies agreed that APD could potentially predict the success of pyeloplasty.^18,19,26,29^ A higher preoperative APD was associated with a higher probability of surgery failure.^15,18^ Despite the presence of studies supporting the effect of APD, 3 studies found that APD had no significant effect on the outcomes of pyeloplasty.^20,21,25^

Then, literature has shown the relationship between cortical thickness and the results of pyeloplasty (Table 3).^1,23,26^ A study stated that parenchymal thickness of ≤0.75 cm was the predictor correlated with renal function deterioration after a pyeloplasty.^23^ However, this contrasts with the other 2 studies, which stated that cortical thickness has no effect.^21,25^ Han et al^20^ found that patients with a higher preoperative hydronephrosis area-to-renal parenchyma area (HARP) are expected to show decreased postoperative renal function.

Delayed tissue tracer transit (TTT) can predict renal function improvement after pyeloplasty (Table 4). Tissue tracer transit or cortical transit can be defined as the period when the cortical rim is still visible, and practically no subcortical areas activities.^30^ Song et al^25^ showed the chance of renal function improvement of >5% in patients with prior DRF of <45% is 5.9 times more significant with a delayed TTT. Piepsz et al^30^ stated patients with impaired cortical transit had a higher DRF improvement probability following a pyeloplasty. Patients who would benefit from pyeloplasty might be marked by prolonged cortical transit combined with poor kidney washout.

## Discussion

Hydronephrosis is the most often identified genitourinary abnormality on prenatal ultrasounds. In the past, every child who presented with ureteropelvic junction blockage was given the option of corrective surgery. The last 10 years have seen a gradual shift toward observational care of congenital UPJ blockage.

Our systematic review indicates that operative repair in patients with UPJO has the potential for renal function recovery after a pyeloplasty. Experimental studies demonstrated that renal damage due to chronic obstruction depends on the duration of the obstruction and severity.^31^ Patients with impaired kidney function could have a stable or an improved renal function as demonstrated during follow-up with diuretic renography (either increase in GRF or SRF), which suggested that early nephrectomy should be avoided without definitive indications, such as infection or malignancy.^32^ However, no definitive factors could be shown in predicting functional recoverability following a pyeloplasty. In the absence of predictors, other researchers have suggested a urinary diversion trial using an internal double-J stent or an external nephrostomy tube. The intervention could be conducted within 4-8 weeks to find the true chance for recovery. However, management is, in turn, much longer and troublesome for patients.^8,33^ In our systematic review, we evaluated factors that could be predictors for the capability of renal function to recover following a pyeloplasty. From the included studies, several parameters were found to be associated with outcomes of pyeloplasty, namely the age at diagnosis and surgery, APD, preoperative DRF, urinary protein and creatinine ratio, cortical thickness, SFU, TTT, and HARP.

Age at surgery was studied in this review as a predictor of pyeloplasty outcome. Logically, the younger the patient during surgery leads to a better outcome; however, a study by Salem et al. suggested that age did not affect the overall likelihood of preoperatively DRF being reduced or improved postoperatively.^22^ This result is probably because renal changes are developmental at birth, which are probably fixed in many patients. This study is also in line with several more studies that showed no difference between the recovery of renal function based on age at diagnosis (pre and postnatal).^19-21^ On the contrary, Nodenstrom et al^18^ showed that patients who were antenatally detected had a better catch-up or improvement in DRF. Onen et al^34^ and Ulman et al^35^ presented cohorts of individuals with an average age at surgery of 5-6.5 months and claimed DRF recovery after pyeloplasty compared to the mean age of 20 months; however, they did not report how many patients had a DRF improvement of >5.

The next predictor was baseline DRF. Harraz et al^21^ showed that improved postoperative DRF was expected in patients with a preoperative DRF of ≤40%, and this result had been reported previously in adult and pediatric cohorts.^1^ With less initial DRF, the room for improvement of DRF is also greater. Almodhen et al^36^ revealed that no patients with a baseline DRF of more than 45% achieved DRF recovery of over 5% compared to those with under 45% of DRF. Nonetheless, Ortapamuk et al^37^ showed that improvement might not occur, particularly in those with a DRF of under 30%. In the case of failed prior pyeloplasty, the decrease of >5% DRF before and after pyeloplasty contributes to the lower chance of functional recovery after redo-pyeloplasty, suggesting that redo-surgery should be performed before DRF has severely deteriorated.^38^ Cortical thickness has been shown to be a significant factor affecting recovery. The healthy nephrons might explain the capability of recovery by the kidney.^21,38^

We also assessed the impact of ultrasound evaluation as the predictor factor. Chipde et al^27^ found that APD was the most crucial predictor of post-pyeloplasty renal function improvement. A higher preoperative APD was associated with a higher probability of surgery failure. A study by Kandur et al^39^ has shown that a 20 mm APD threshold indicated a severe obstruction and low DRF threshold. Another study also revealed that a 40-mm APD could be a risk of failure in patients receiving a laparoscopic approach.^19^ Society for fetal urology hydronephrosis grade was also one of the factors associated with pyeloplasty outcomes. In children with congenital hydronephrosis, SFU grade has been shown to be correlated with renal function, in which a higher grade indicates decreased DRF preservation.^40^ Patients with a higher preoperative SFU grade had a lower postoperative improvement than those with more postoperative improvement.^5^ Ultrasound evaluation of both cortical thickness and APD measurement in combination with a dynamic renal scan should be the best diagnostic workup and could not replace one another.^18,27^

Obstruction of the kidney due to UPJ causes a substantial delay in the 99mTc-MAG3 washout, shown as delayed TTT. The renal pelvis’ elevated pressure causes declined GFR, and to balance the GFR and filtration fraction, the renin–angiotensin system will then be activated. Nephrons sclerosis and decreased renal function may be caused by chronic activation of the system.^41,42^ A study by Son et al^25^ showed that the statistically significant predictor of improved renal function by over 5% in individuals with a DRF of under 45% was delayed TTT. Piepsz et al^30^ also suggested that prolonged cortical transit duration could provide benefits for a pyeloplasty and with a high chance of DRF improvement. These results suggested that individuals with delayed TTT and decreased DRF should be assessed to undergo surgical repair immediately.

The last factor investigated was the urinary biochemical parameter. This factor can be used to overcome the limitation of ultrasonography and renography. Nowadays, there are several other urine biomarkers that can be used as explained in the previous systematic review.^28^ As these markers were already review in previous article, we did not compare them further in this article. In this review, we found a simple laboratory evaluation that can be used in remote or rural hospitals. A study by Beharrie et al^43^ compared the samples taken from above and below the obstruction. The results suggested a statistically significant difference between the renal pelvis’ and bladder’s (protein–creatinine ratio (Pr/Cr).^43^ This indicates that severe chronic obstruction altering renal function could present as biochemical parameter changes, including sodium fractional extraction, creatinine clearance, and protein excretion leading to proteinuria. Renal dysplasia may be associated with UPJO; furthermore, changed biochemical parameters, such as proteinuria, can be utilized to predict the result of obstruction release. Chipde et al^27^ suggested that all individuals with a preoperative Pr/Cr of less than 0.5 showed improved renal function after pyeloplasty. This result may be beneficial in situations with limited resources, where ultrasound and renal urography are not available; however, more studies are needed to determine the cutoff and reliability.

Most of the included studies, which were retrospective, and the heterogeneous characteristics of the study were considered limitations of this study. The nuclear tests for renal function varied between MAG3, DMSA, and DTPA might cause varied results. The included studies’ quality was also average based on the design, methodology, and bias risk. Ideally, the next studies should standardize the tools for a gold standard renal function evaluation.

Our systematic review supported that there were several factors associated with the outcome of pyeloplasty. The suggested predictor was the combination of ultrasound evaluation, such as APD and cortical thickness and DRF evaluation prior to surgery. Urinary biomarkers such as protein creatinine ratio taken from the renal pelvis could be beneficial in limited resources, but more studies are required to support this.

## Figures and Tables

**Table 1.** Studies’ Characteristics

Author, Year	Study Design	Country	Sample Size (n)	Age	Gender	Renal Function Test	Surgery Side	Procedure	Surgery Type
El-Desoukey et al^26^, 2021	Prospective	Egypt	30	Mean 8.85 ± 4.9 years	Male 18 Female 12	GFR and DRF	N/A	Anderson-Hynes dismembered pyeloplasty	Open
Guler et al^19^, 2021	Retrospective	Türkiye	51	Mean 59 ± 49.8 months	Male 36 Female 15	RRF, DRF, and MAG3 excretion.	Right 17 Left 34	Anderson-Hynes dismembered pyeloplasty	Laparoscopic
Han et al^20^, 2020	Retrospective	Korea	187	Mean 3.0 ± 4.8 years	Male 147 Female 45	DRF	Right 45 Left 142	Anderson-Hynes dismembered pyeloplasty Fenger plasty (2 patients)	Open 147 Laparoscopic 28 Robot-assisted laparoscopic 12
Nordenstrom et al^18^, 2020	Prospective	Sweden	83	Range 0-16 years	Male 63 Female 22	DRF and eGFR	Right 40 Left 43	Anderson-Hynes dismembered pyeloplasty	N/A
Song et al^25^, 2017	Retrospective	Korea	126	Median 9 (range 1-216) months	Male 99 Female 27	DRF	Right 36 Left 90	N/A	Open 107 Laparoscopic 16 Robotic 3
Chung et al^30^, 2014	Retrospective	Korea	18	Mean 55.50 ± 47.15 months	Male 15 Female 3	DRF	Right 2 Left 16	Anderson-Hynes dismembered pyeloplasty: 13 No dismembered: 4	N/A
Harraz et al^21^, 2013	Retrospective	Egypt	196	Median 4 (range 0.2-18) years	Male 147 Female 49	DRF and eGFR	Right 78 Left 118	Anderson-Hynes dismembered pyeloplasty	Open
Helmy et al^28^, 2013	Prospective	Egypt	68	Mean 3.6 (range 0.1-12) years	Male 35 Female 33	DRF	Right 25 Left 43	Anderson-Hynes pyeloplasty	N/A
Chipde et al^27^, 2012	Prospective	India	52	Mean 4.26 years (3 months – 18 years)	Male 34 Female 18	DRF	N/A	Anderson-Hynes dismembered pyeloplasty	Laparoscopic
Piepsz et al^29^, 2011	Prospective	Belgium	16	Median 18 month	N/A	DRF	N/A	Anderson-Hynes dismembered pyeloplasty	Open
Salem et al^22^, 1995	Retrospective	US	98	Median 1 year (5 days to 16 years)	Male 65 Female 33	DRF and eGFR	Right 60 Left 48	Anderson-Hynes dismembered pyeloplasty	Open

DRF, differential renal function; GFR, glomerular filtration rate; RRF, relative renal function.

**Table 2.** Differential Renal Function as Predictive Factor

Author, Year	Follow-up Period	Modalities	Predictive Factors	Interpretation	Non-significant Factors
El-Desoukey et al^26^, 2021	1, 3, 6 months	DTPA renogram	T1/2 (*P *= .001) GFR (*P *= .001) Split function (*P *= .001)	T1/2 postoperatively were significantly lower among the improved cases, while GFR, and split function were significantly higher among the improved cases.	N/A
Nordenstrom et al^18^, 2020	3 and 18 months	Renal scan (MAG-3)	Preoperative DRF (OR = 0.90, 95% CI 0.84-0.97, *P *= .0045)	Low preoperative DRF predict improvement in DRF	High APD/renal length ratio High APD/parenchymal thickness ratio
Chung et al^30^, 2014	Mean 44.83 ± 28.86 months	Renal scintigraphy	dDRF (*P *< .001) dRCT (*P *< .0001)	A severely decreased renal function following the initial surgery could severely influence the likelihood of recovering the initial renal function after a redo of pyeloplasty.	Baseline DRF
Harraz et al^21^, 2013	Median 12 months (6-91 month)	Diuretic scintigraphy Ultrasound	Baseline DRF Max cortical thickness	Baseline DRF with less than 40% function was most likely to improve.	AP Diameter (*P *= .11) Diagnosis time pre vs. ante natal (*P *= .42)
Salem et al^22^, 1995	1.5 years (1-5 years)	Ultrasound Excretory urography Abdominal CT scan	Base DRF	64% patients with Base DRF 20%-40% show at least 5% improvement compared to 11% patients with > 40% DRF and 56% of patients with < 20% DRF	Age at the time of surgery (*P *= .960)

APD, anterior posterior diameter; DRF, differential renal function; GFR, glomerular filtration rate; OR, odds ratio; UPJO, ureteropelvic junction obstruction.

**Table 3.** Ultrasound Evaluation as Predictive Factor

Author, Year	Follow-Up Period	Modalities	Predictive Factors	Interpretation	Non-Significant Factors
El-Desoukey et al^26^, 2021	1, 3, 6 months	Ultrasound	Pelvic APD (*P *= .001) Parenchymal thickness (*P *= .001)	The pelvic APD postoperatively were significantly lower among the improved cases, while parenchymal thickness was significantly higher among the improved cases.	N/A
Guler et al^19^, 2021	3-6 months	USG and MAG-3 diuretic renal scan	APD of renal pelvis (OR = 0.296, 95% CI 0.002-0.775, *P *= .045)	A risk factor for decreased surgical success was a larger preoperative APD.	Age Gender Laterality Etiology of UPJO
Han et al^20^, 2020	Every 3-6 months	USG	HARP (HR 1.740, 95% CI 1.11-2.72, *P *= .015)	Those with preoperative high HARP values were more likely to experience decreased renal function after pyeloplasty	Age (HR 0.84, 95% CI 0.67-1.05, *P *= .133) Gender (HR 2.82, 95% CI 0.87-8.43, *P *= .083) SFU (HR 80.912, *P *= .998) UTD (HR 0.813, *P *= 1.000) APPD (HR 1.02, 95% CI 0.98-1.07, *P *= .38)
Nordenstrom et al^18^, 2020	3 and 18 months	USG and renal scan (MAG-3)	APD (OR 1.1, 95% CI 1.04-1.19, *P *= .0023) Antenatal diagnosis (OR = 0.23, 95% CI 0.06	High anteroposterior diameter and antenatal diagnosis predict improvement in DRF	High APD/renal length ratio High APD/parenchymal thickness ratio
Helmy et al^28^, 2013	At least 6 months	USG	Calyx-to-parenchyma ratio (beta = −1.153, *P *< .001) Anteroposterior diameter (beta = 0.375, *P *= .005) Calyceal dilatation (beta=0.294, *P *= .027)	N/A	Calyceal-pelvis ratio Calyceal length Calyceal width Pelvic-to-cortex ratio
Chipde et al^27^, 2012	3 and 6 months	Ultrasound	APD (*P *= .018) PCR (.038)	The APD and PCR were significantly lower among improved cases	Maximal cortical thickness

APD, anterior posterior diameter; APPD, anterior to posterior pelvic diameter; DRF, differential renal function; HARP, hydronephrosis area-to-renal parenchyma ratio; HR, hazard ratio; OR, odds ratio; PCR, Pelvic cortical ratio; UPJO, ureteropelvic junction obstruction; UTD, urinary tract dilation.

**Table 4.** Main Findings of Other Predictive Factors

Author, Year	Follow-up Period	Modalities	Predictive Factors	Interpretation	Non-significant Factors
Song et al^25^, 2017	Median 35.3 (range 3-173) months	Tc-MAG3 diuretic renography	Delayed TTT (OR 14.25, 95% CI 2.13-95.21, *P *= .006)	The only independent predictor of improved renal function (≥10% improvement) was delayed TTT.	Gender (OR 0.47, 95% CI 0.06-3.20, *P *= .441) Age (OR 1.01, 95% CI 0.98-1.03, *P *= .482) Laterality (OR 0.67, 95% CI 0.12-3.66, *P *= .645) Prenatal hydronephrosis diagnosis (OR 14.21, 95% CI 0.03-6714.3, *P *= .398) Preoperative pain (OR 2.96, 95% CI 0.00-2771.5, *P *= .756) SFU (OR 0.47, 95% CI 0.03-4.76, *P *= .470) Preoperative APPD (OR 0.91, 95% CI 0.45-1.83, *P *= .802) Cortical thickness (OR 0.27, 95% CI 0.16-4.72, *P *= .376)
Chipde et al^27^, 2012	3 and 6 month	Diuretic renogram	Pelvic urine Pr/Cr	Urine Pr/Cr taken form renal pelvic were significantly lower in improved cases	Difference in bladder pr/Cr
Piepsz et al^29^, 2011	Median 37 months	MAG3 renogram	Cortical transit time	Prolonged cortical transit and poor whole kidney washout show a higher probability of DRF post-operative improvement	N/A
Salem et al^22^, 1995	1.5 years (1-5 years)	Ultrasound Abdominal CT scan	Clinical presentation	Symptomatic patients (abdominal mass, pain, urinary tract infection) show better improvement than incidental (totally incidental finding to the prenatal US)	Age at the time of surgery (*P *= .960)

APPD, anterior to posterior pelvic diameter; OR, odds ratio; SFU, society for fetal urology; TTT, tissue tracer transit; Pr/Cr, protein–creatinine ratio.
